# The Role of the Gut, Urine, and Vaginal Microbiomes in the Pathogenesis of Urinary Tract Infection in Women and Consideration of Microbiome Therapeutics

**DOI:** 10.1093/ofid/ofae471

**Published:** 2024-08-19

**Authors:** Amal Naji, Drew Siskin, Michael H Woodworth, John R Lee, Colleen S Kraft, Nirja Mehta

**Affiliations:** Piedmont Hospital, Atlanta, Georgia, USA; Emory University, Atlanta, Georgia, USA; Division of Infectious Diseases, Department of Medicine, Emory University, Atlanta, Georgia, USA; Division of Nephrology and Hypertension, Weill Cornell Medicine, New York, New York, USA; Division of Infectious Diseases, Department of Medicine, Emory University, Atlanta, Georgia, USA; Department of Pathology and Laboratory Medicine, Emory University, Atlanta, Georgia, USA; Division of Infectious Diseases, Department of Medicine, Emory University, Atlanta, Georgia, USA

**Keywords:** bacteria, gut, microbiome, urinary, vaginal

## Abstract

The gut, urine, and vaginal microbiomes play significant roles in the pathogenesis of recurrent urinary tract infections (rUTIs). Analysis of these microbiota has shown distinct associations with urinary tract infections. Encouraging data indicate that rUTIs may be responsive to microbiome treatments such as fecal microbiota transplantation, expanding potential treatments beyond antibiotics, hydration, and behavioral interventions. If successful, these nonantibiotic therapies have the potential to increase time between rUTI episodes and reduce the prevalence of multidrug-resistant organisms. In this review, we discuss the role of the 3 microbiomes in the pathogenesis of rUTI and utilization of live biotherapeutic products as therapy for rUTI.

## Teaser

This review discusses the role of the gut, urine and vaginal microbiome on recurrent urinary tract infection pathogenesis, as well as how live biotherapeutic products such as fecal microbiota transplantation can influence the microbiome and affect clinical outcomes

Recurrent urinary tract infections (rUTIs) increase morbidity and diminish quality of life due to their recurrent nature, impact on sexual relationships, and chronic discomfort [1, 2]. These infections are typically defined as at least 2 episodes of UTIs within 6 months or 3 episodes within 12 months [3]. Numerous approaches have been explored to identify mechanisms that perpetuate rUTIs that may be amenable to therapeutic development. In 2008, the Human Microbiome Project (HMP) was launched to understand the contribution of microbial composition to human health and disease [4]. Initially, the urinary system was not included in this project because it was thought to be sterile. However, the development of improved culturing methods revealed that the urinary system harbors a distinct microbiome [4, 5].

The pathogenesis of UTIs begins with the introduction of virulent bacteria of the urinary system, referred to as uropathogens [6]. Most uropathogens are of gastrointestinal origin; however, data also demonstrate an important role of the vaginal and urine microbiomes in the development of recurrent UTIs [7–9]. A large study of 435 cultured urine specimens found that approximately two-thirds of these bacteria were shared with the human gut repertoire, and one-third with the human vaginal repertoire. This finding underscores the important contributions of both populations to the commensal bacteria of the urine microbiome in women [7]. Given the rising prevalence of antibiotic-resistant organisms (a subset of which are known as multidrug-resistant organisms [MDROs]) and limited treatment options, researchers have focused on finding novel strategies to treat recurrent UTIs [10]. This review will describe existing data regarding the interplay of the gut, urine, and vaginal microbiomes in driving rUTIs and the impact of microbiota therapeutics such as fecal microbiota transplantation (FMT) on the frequency and causative pathogens of urinary tract infections and the antibiotic susceptibility of the various uropathogens implicated in UTIs.

## METHODS

We conducted a PubMed query by searching for keywords including “gut microbiome,” “urine microbiome,” “vaginal microbiome,” “recurrent urinary tract infection,” and “antibiotic resistant urinary tract infection.” Our search yielded 523 papers. Two authors (A.N. and D.S.) reviewed the papers, then filtered them to include papers that highlight the role of gut, urine, and vaginal microbiomes, and their potential role in urinary tract infections. We included only human studies on populations greater than 18 years of age. At this point, only 40 studies remained. These 40 papers included systematic reviews, literature reviews, retrospective studies, case reports, and prospective/retrospective case studies.

### Gut Microbiome and Urinary Tract Infections

The gut bacteria play an important role in urinary tract infections. Several studies have evaluated the impact of the competitive ecosystem in the gut on the thriving of uropathogens such as *Enterobacteriaceae* [11–13]. In a study by Magruder et al., the prevalence of *Faecalibacterium* and *Romboutsia*, regarded as commensal bacterial taxa, was significantly elevated in the kidney transplant recipients who did not develop *Enterobacteriaceae* bacteriuria as compared with kidney transplant recipients who did [11]. In a case–control study of children, the gut abundance of *Enterobacter* spp. in children with febrile UTIs was higher than healthy children, specifically with a higher relative abundance of *Escherichia coli* in patients with UTIs [14].

Several different mechanisms may explain the function of commensal bacteria in resisting uropathogen gut colonization, such as direct production of bacteriocin, depletion of nutrients, and production of short-chain fatty acids (SCFAs) [12]. The production of the peptide bacteriocin confers a bactericidal effect on pathogenic bacteria such as *Enterobacteriaceae*. It inhibits cell wall synthesis and disrupts the bacterial membrane by forming pores [15]. Additionally, SCFAs, a product of gut fermentation, often lead to an acidic intracellular medium. The SCFA-mediated acidic milieu inhibits the replication of uropathogenic organisms such as *E. coli, Klebsiella pneumoniae,* and other members of the *Enterobacteriaceae* by decreasing the availability of oxygen and nitrate. SCFAs were also found to stabilize hypoxia-inducible factor–1 (HIF-1), thus increasing production of antimicrobial proteins. The decrease in the abundance of these organisms in the intestines secondary to the production of bacteriocin and SCFA decreases the risk of bacteriuria and likely urinary tract infections [12].

### Fecal Microbiota Transplantation and Recurrent Urinary Tract Infections

Given the strong relationship between the gut microbiome and etiologic agents of UTIs, several studies have evaluated the use of FMT in the treatment of rUTIs [16–18]. FMT increases the alpha diversity of the gut, reducing the relative abundance of uropathogens, and has also been shown to reduce urinary tract infections [19]. However, no consensus has been established regarding the routine utilization of FMT to treat rUTIs in general or UTIs due to multidrug-resistant organisms, and the Food and Drug Administration (FDA) has not approved the use of FMT in the treatment of urinary tract infections [20]. Most studies published regarding the use of FMT in the treatment of UTIs are case reports and case series (Table 1) [16–18, 21–27]. Most of these studies have shown complete resolution of UTI symptoms and a decrease in the prevalence of uropathogens for up to 1 year after FMT. Moreover, a study by Tariq et al. reported a significant 4-fold reduction in the frequency of urinary tract infections 1 year after FMT. An additional study by the same group also reported a reduction in UTIs after patients were treated with FMT for recurrent *Clostridioides difficile* infection. These reductions were largely attributed to the ability of FMT to replenish the healthy gut microbiome, thus replacing uropathogens and decreasing the risk of UTIs [17, 28]. None of these studies reported severe or life-threatening adverse events, thus suggesting that FMT could be a safe decolonization modality [16–18, 21–24].

**Table 1.** Fecal Microbiota Transplantation in the Treatment of Urinary Tract Infections

Authors	Patients’ Characteristics	Type of Study	Primary End Point	Outcome	Adverse Events
Aira et al., 2020 [21]	93-year-old female with history of recurrent UTIs, chronic hepatitis C, end sigmoid colostomy for acute diverticulitis, and recurrent *C. difficile*; 3 UTIs in the past year	Case report	Efficacy of FMT in treatment of recurrent *C. difficile* infection	No symptoms of *C. difficile* infection after a year No new symptomatic UTIs after 1 year Reduction in *Enterobacteriaceae* in gut post-FMT	None
Jeney et al., 2020 [16]	Ten women with recurrent refractory urinary tract infections	Prospective case series	Comparison of number of UTIs 6 months before enema-based FMT with number of UTIs 6 months post-FMT	Nonsignificant decrease in the number of symptomatic UTIs; out of 10, 4 women no longer met criteria for rUTIs, and 3 had no further UTIS	None
Ramos-Martinez et al., 2020 [22]	43-year-old patient with recurrent *C. difficile* infection and rUTI	Case report	Efficacy of FMT in treatment of recurrent *C. difficile*	No new episodes of *C. difficile* or UTI 10 months post-FMT	None
Hocquart et al., 2019 [23]	73-year-old woman with irritable bowel syndrome and recurrent urinary tract infections	Case report	Efficacy of FMT in reducing frequency of stools and improving stool consistency	No recurrence of UTIs; decrease in IBS symptoms after FMT	None
Biehl et al., 2018 [18]	43-year-old kidney transplant recipient with recurrent UTIs	Case report	Efficacy of FMT in treatment of recurrent UTIs	No new UTI symptoms 9 months after FMT; decrease in *Enterobacteriaceae* over time	None
Wang et al., 2018 [24]	83-year-old woman with 25-year history of recurrent urinary tract infections	Case report	Efficacy of FMT in treatment of recurrent *C. difficile* infections and recurrent UTIs	Complete resolution of UTI and *C. difficile* infection 9 months after FMT	None
Stalenhoef et al., 2017 [25]	34-year-old woman with Verona integron–encoded metallo-β-lactamase-induced recurrent urinary tract infections	Case report	Efficacy of FMT in reduction of MDR stains in stool	Persistence of ESBL- producing *E. coli* in stool after FMT No significant difference in microbial diversity after FMT	Loose stools for 3 days
Staley et al., 2017 [26]	19 patients who underwent FMT for treatment of recurrent *C. difficile* infection	Case series	Efficacy of aminoglycoside-based outpatient UTI treatment regimen in	Resolution of UTI symptoms No recurrence of *C. difficile*	Tenderness over gluteal muscles
…	…	…	preservation of gut integrity	No significant difference in alpha diversity of gut after treatment with aminoglycosides	…
Tariq et al., 2017 [17]	8 patients with recurrent UTIs who underwent FMT	Retrospective case series	Efficacy of FMT in reducing the frequency of UTIs cause by MDRO	4-fold reduction in the median frequency of UTIs post-FMT No recurrence of *C. difficile* infection at 1 year post-transplant Decrease in frequency of MDR organisms causing UTIs	None
Singh et al., 2014 [27]	60-year-old male post–renal transplant with recurrent pyelonephritis caused by ESBL-producing *E. coli*	Case report	Efficacy of FMT in decolonizing patient from ESBL	Negative rectal cultures for ESBL at 12 weeks post follow-up No symptoms of UTI	Mild diarrhea and abdominal cramps

Abbreviations: ESBL, extended-spectrum beta-lactamase; FMT, fecal microbiota transplantation; IBS, irritable bowel syndrome; MDRO, multidrug-resistant organism; UTI, urinary tract infection.

### Fecal Microbiota Transplantation and Antibiotic Susceptibility

The emergence of MDROs has been a major public health concern [29–31]. The gut has been identified as a reservoir for MDROs, constituting the “resistome” for both pathogenic and nonpathogenic bacteria [29, 32]. Intestinal colonization by these organisms was found to precede several invasive infections including rUTI, thus contributing to an increase in morbidity and mortality [33]. Therefore, gut decolonization through FMT has been proposed as a promising approach to diminish such infections. The eradication of MDROs occurs by increasing the phylogenetic diversity of the gut microbiome with an increase in the frequency of commensal bacteria and decrease in the frequency of pathogenic bacteria such as *Enterobacteriaceae* [30, 31].

Recently, several studies have evaluated the safety and efficacy of FMT in eradicating MDRO colonization, with somewhat mixed results [34–36]. A study by Seong et al. demonstrated that patients undergoing FMT were found to have a 68.6% decolonization rate of carbapenemase-producing *Enterobacteriaceae* (CPE) and vancomycin-resistant enterococci (VRE) within a year after transplant compared with only 27.1% of patients who did not receive an FMT [34]. This was shown to be inconsistent with data reported in a systematic review by Yoon et al., which showed a nonsignificant difference in the eradication rates of the various MDROs such as CPE, VRE, and extended spectrum beta-lactamase (ESBL)–producing *Enterobacteriaceae* [35]. Similarly, Stalenhoef et al. attributed the nonsignificant reduction in the UTI episodes to the failure of FMT to clear ESBL *E. coli* [25]. A recent randomized controlled trial (RCT) investigating the role of FMT in MDRO decolonization in renal transplant recipients demonstrated that FMT can potentially support bacterial strain replacement of resistant organisms [37]. Hyun et al. found that FMT downregulated various resistance genes including *vanA* and *blaKPc,* thus enhancing antibiotic susceptibility [38]. The success of FMT in rUTIs has been associated with several factors, including, but not limited to, treatment with antibiotics before or after FMT, frequency of FMT, host characteristics, and characteristics of the MDROs [35, 39–41]. However, no concrete recommendations for FMT administration for rUTIs have been established to date. It is worth noting that studies have demonstrated acquisition of donor-derived antimicrobial-resistant genes through FMT, emphasizing the importance of developing appropriate screening programs to identify healthy stool donors in the success of FMT [42–44]. However, Leung et al. reported the acquisition of donor-derived antimicrobial-resistant genes through FMT. This further emphasizes the importance of developing appropriate screening programs to identify healthy stool donors in the success of FMT [44].

Limited studies evaluating the efficacy of FMT in the eradication of MDROs in in vivo models exist in the literature [45, 46]. In a study by Mrazek et al., murine models infected with multidrug-resistant *Pseudomonas aeruginosa* underwent FMT from either human or mouse fecal donors. FMT, irrespective of the source of the donated fecal samples, significantly decreased the load of MDROs [45]. The introduction of commensal bacteria allows for clearance of MDROs at different rates [46].

### Urine Microbiome

The urinary tract was not included in early microbiome research because urine was thought to be naturally sterile [10, 47, 48]. This hypothesis has since been rejected by the introduction of high-throughput sequencing. Acknowledging the technical limitations of sequencing low-biomass specimens and potential contamination, urine sequencing analyses have detected a wide diversity of bacteria in the urine of asymptomatic healthy individuals [6, 8]. The urinary tract has been shown to host a complex microbial community. This community includes core bacterial genera, which are often altered by diseases and infections. In general, both female and male urine microbiomes have been found to be dominated by the phylum Firmicutes [48]. To date, what most studies have focused on analyzing is the female urine microbiome due to the higher incidence of urological disorders in women. Females have been found to have a heterogenous mix of bacterial genera compared with males [48–50]. An analysis of catheterized urine samples from premenopausal and postmenopausal women showed that the samples were largely dominated by the *Lactobacillus* genus. Other bacterial genera isolated included *Gardnerella, Prevotella, Escherichia-Shigella, Atopobium, Streptococcus,* and *Dialister*. The variation in the bacterial composition of the urine microbiome is often determined by multiple factors, including age, ethnicity, sexual activity, and hormonal changes [49].

The urine microbiota profile often plays a substantial role in predicting the risk of urinary tract disorders. The relative abundance of certain bacterial taxa has been found to predict future risk of bladder dysbiosis. For example, women with a lower relative abundance of *Lactobacillus* were found to have an increased risk of bladder disorders including urinary incontinence [49]. Moreover, a disruption in the diversity of the urine microbiome is implicated in the pathogenesis of rUTIs [8]. This occurs via various mechanisms including migration of resistant uropathogenic bacteria from the gut, re-infection from an external source, or bacteria persistent in the urothelium [16]. Taxonomic profiling of the various human microbiomes has shown a significant overlap between the urine, gut, and vaginal microbiomes. Dubourg et al. identified bacteria in the urine using the 16S rRNA gene sequencing method. Of those bacterial species, 64.1% were found to be shared with the human gut microbiome. Therefore, the origin of bacterial constituents of the urine microbiome was found to be mostly of gastrointestinal origin [6].

While antibiotic therapy remains the mainstay treatment for UTIs, conventional antibiotics often cause long-term modifications in the urine microbiome, recurrent cystitis, and rise in MDROs [10, 51]. Then, these organisms frequently persist in the bladder by formation of intracellular bacterial communities, typically within a biofilm-like structure. Biofilms can further contribute to antibiotic resistance by degrading antibiotics or utilizing beta-lactamases. In the elderly and immunocompromised population, the uropathogenic reservoirs are commonly polymicrobial; therefore, inappropriate antibiotic intervention can have detrimental effects on resistance [10, 51, 52].

### Urine Virome

An analysis of the viral constituents of the urinary tract revealed the essential role that lytic phages play in treating multidrug-resistant organisms (MDROs). This effectiveness is largely attributed to the production of lytic proteins, including endolysins and virion-associated peptidoglycan hydrolases, which lead to the lysis of bacterial cell walls [10].

### Urine Mycobiome

Research on microbial communities associated with urinary tract infections has predominantly focused on studying bacteria. Consequently, nonbacterial components of the urinary system have been largely understudied. Previously, there was a misconception that these organisms play a minor role in the development of urinary tract infections. Among the fungi detected in urine, species from the *Saccharomyces* class, such as *Saccharomyces* and *Candida*, were identified [53, 54]. Studies have demonstrated that changes in the host's immune state can significantly contribute to fungal colonization and pathology in the urinary tract system. The urinary mycobiome in other diseases, such as interstitial cystitis, has been better characterized and is being increasingly appreciated in its role for other urologic disorders [55].

### Vaginal Microbiome

While the gut is most considered the reservoir for enteropathogens leading to UTI, increasing research demonstrates that the vaginal microbiome also plays an essential role in the pathogenesis of UTIs in women [9, 56, 57]. Over 460 species of bacteria have been described in vaginal microbiome studies [58]. The urine microbiome shares up to one-third of microbial species with the vaginal microbiome. Increasing data demonstrate that the pathogenesis of recurrent UTIs is influenced by the vaginal microenvironment in addition to the gut microenvironment [59]. When the vaginal microbiome is disturbed or altered, the risk for UTIs increases [9, 60]. Sexual activity and douching are some of the strongest independent risk factors for the development of UTIs in women, further demonstrating the close link between the vaginal and urinary microbiota and implicating frequent mechanical transfer of bacteria between vaginal and urinary mucosal sites [61]. *Lactobacillus*, the most common species in the vaginal microbiome in women of reproductive age, produces bacteriocins and hydrogen peroxide that protect against colonization of various pathogens, including *E. coli* [62]. Dysbiosis of the microbiome and decreased population of *Lactobacillus* have been implicated in other disease states such as bacterial vaginosis and *Neisseria gonorrhoeae* infection, in addition to higher rates of UTI [57, 62, 63].

The role of the vaginal microbiome in the facilitation of *E. coli* colonization and pathogenesis of the bladder has been noted in several studies. In a study of 77 women, *E. coli* strains from the vagina and urine were 99.72% similar by whole-genome pairwise average nucleotide identity [64]. Patients with a history of UTI have been found to have higher levels of *E. coli* colonization in the vagina and urine compared with patients without a history of UTI [65]. Additionally, commensal vaginal bacteria such as *Gardnerella vaginalis* may transiently enter the bladder, leading to egress from latent bladder reservoirs, leading to UTI in murine models [61]. *Gardnerella vaginalis* and Group B *Streptococcus* have been implicated in a process termed “covert pathogenesis,” by which the transient presence of these organisms early in infection leads to immunomodulation, which may increase susceptibility to clinical infection by more traditional uropathogens such as *E. coli* [57, 61]. Additionally, the hormone changes during menopause change the vaginal microbiome and decrease the amounts of *Lactobacillus* species, increasing rates of rUTIs and UTIs [60]. Topical estrogen has long been recommended to postmenopausal women with recurrent UTIs, with multiple studies demonstrating lower UTI rates in patients after estrogen use [66]. In a large cohort study of postmenopausal women, vaginal colonization with *E. coli* was more frequent in women without estrogen replacement [67]. Estrogen replacement leads to higher levels of *Lactobacillus* in this population, thereby creating a microbiome that is more resistant to colonization by uropathogens [64, 67].

While microbiome therapeutics are common in the gut microbiome with FMT, manipulation of the vaginal microbiome is less well studied. In a double-blind placebo- controlled trial that enrolled 100 premenopausal women with at least 1 UTI in the past 12 months, patients who received *Lactobacillus crispatus* suppositories, a species that produces high quantities of H_2_O_2_, demonstrated that higher quantities of *L. crispatus* colonization were associated with lower levels of rUTI [60]. Moreover, the use of vaginal and oral probiotics has been shown to have a promising role in decreasing recurrent urinary tract infections in women. They have been shown to halt the ascension of uropathogens by interfering with pathogen adhesion, biofilm formation, and expression of virulence factors. They were also shown to modulate the host's defense against uropathogens [68]. Given these results, further studies evaluating the role of fortification of the vaginal microbiome in preventing UTIs are needed. At present, the focus exclusively on the gut microbiome (using FMT and other microbiome products) may only be addressing part of the full mechanism driving rUTIs.

### Interaction Between Urine, Gut, and Vaginal Microbiomes

The urine, vaginal, and gut microbiomes are interconnected and interact with one another. The exchange and interaction of organisms between these microbiomes contribute to urinary tract infections. Bacteria from the gut are influential in infections occurring higher up in the urinary system, whereas bacteria from the vaginal microbiome are often involved in infections in the lower urinary tract. Hence, identifying factors that influence these microbiomes is crucial in efforts to reduce the incidence of urinary tract infections [9].

## CONCLUSIONS

The gut, urine, and vaginal microbiomes play an important role in the pathogenesis of UTIs and rUTIs. Historically, antibiotics were the sole treatment for managing urinary tract infections. However, the emergence of multidrug-resistant organisms has prompted exploration of more innovative approaches, such as vaginal probiotics and fecal microbiota transplant. These treatments have shown promising results and hopefully will offer effective alternatives in combating urinary tract infections, especially in cases where traditional antibiotic therapies may be less effective due to resistance issues.
